# Deep learning-based image quality assessment: impact on detection accuracy of prostate cancer extraprostatic extension on MRI

**DOI:** 10.1007/s00261-024-04468-5

**Published:** 2024-07-03

**Authors:** Yue Lin, Mason J. Belue, Enis C. Yilmaz, Yan Mee Law, Katie M. Merriman, Tim E. Phelps, David G. Gelikman, Kutsev B. Ozyoruk, Nathan S. Lay, Maria J. Merino, Bradford J. Wood, Sandeep Gurram, Peter L. Choyke, Stephanie A. Harmon, Peter A. Pinto, Baris Turkbey

**Affiliations:** 1 Molecular Imaging Branch, National Cancer Institute, National Institutes of Health, 10 Center Dr., MSC 1182, Building 10, Room B3B85, Bethesda, MD 20892 USA; 2 Department of Radiology, Singapore General Hospital, Singapore, Singapore; 3 Laboratory of Pathology, National Cancer Institute, National Institutes of Health, Bethesda, MD USA; 4 Center for Interventional Oncology, National Cancer Institute, National Institutes of Health, Bethesda, MD USA; 5 Department of Radiology, Clinical Center, National Institutes of Health, Bethesda, MD USA; 6 Urologic Oncology Branch, National Cancer Institute, National Institutes of Health, Bethesda, MD USA

**Keywords:** Prostatic neoplasms, Radiology, Magnetic resonance imaging, Image quality, Artificial intelligence

## Abstract

**Objective:**

To assess impact of image quality on prostate cancer extraprostatic extension (EPE) detection on MRI using a deep learning-based AI algorithm.

**Materials and methods:**

This retrospective, single institution study included patients who were imaged with mpMRI and subsequently underwent radical prostatectomy from June 2007 to August 2022. One genitourinary radiologist prospectively evaluated each patient using the NCI EPE grading system. Each T2WI was classified as low- or high-quality by a previously developed AI algorithm. Fisher’s exact tests were performed to compare EPE detection metrics between low- and high-quality images. Univariable and multivariable analyses were conducted to assess the predictive value of image quality for pathological EPE.

**Results:**

A total of 773 consecutive patients (median age 61 [IQR 56–67] years) were evaluated. At radical prostatectomy, 23% (180/773) of patients had EPE at pathology, and 41% (131/318) of positive EPE calls on mpMRI were confirmed to have EPE. The AI algorithm classified 36% (280/773) of T2WIs as low-quality and 64% (493/773) as high-quality. For EPE grade ≥ 1, high-quality T2WI significantly improved specificity for EPE detection (72% [95% CI 67–76%] vs. 63% [95% CI 56–69%], *P* = 0.03), but did not significantly affect sensitivity (72% [95% CI 62–80%] vs. 75% [95% CI 63–85%]), positive predictive value (44% [95% CI 39–49%] vs. 38% [95% CI 32–43%]), or negative predictive value (89% [95% CI 86–92%] vs. 89% [95% CI 85–93%]). Sensitivity, specificity, PPV, and NPV for EPE grades ≥ 2 and ≥ 3 did not show significant differences attributable to imaging quality. For NCI EPE grade 1, high-quality images (OR 3.05, 95% CI 1.54–5.86; *P* < 0.001) demonstrated a stronger association with pathologic EPE than low-quality images (OR 1.76, 95% CI 0.63–4.24; *P* = 0.24).

**Conclusion:**

Our study successfully employed a deep learning-based AI algorithm to classify image quality of prostate MRI and demonstrated that better quality T2WI was associated with more accurate prediction of EPE at final pathology.

**Supplementary Information:**

The online version contains supplementary material available at 10.1007/s00261-024-04468-5.

## Introduction

Beyond its role in detecting clinically significant prostate cancer, multiparametric MRI (mpMRI) plays an important role in preoperative local staging, particularly in depicting extraprostatic extension (EPE). EPE is a significant indicator of prostate cancer aggressiveness and is associated with a higher likelihood of positive surgical margins, increased rates of biochemical recurrence, and decreased overall survival following radical prostatectomy (RP) [1]. Early detection of EPE on mpMRI can influence the choice of treatment and surgical approach, minimizing post-operative complications [2, 3]. Studies have suggested that mpMRI might outperform traditional clinical risk calculators in predicting pathological EPE [4, 5]. Furthermore, integrating mpMRI with clinical risk assessments enhances the accuracy of predicting pathological EPE [6]. Thus, the National Cancer Institute (NCI) EPE grading system was established to standardize and to help improve EPE prediction using T2-weighted imaging (T2WI) [7].

Despite its growing value in prostate cancer diagnostic workup, mpMRI's role in local staging has faced challenges due to its moderate sensitivity and positive predictive value [8]. These limitations are further exacerbated by high variability in acquisition parameters across centers, which can affect overall image quality and ultimately reader interpretation and diagnostic performances [9]. The Prostate Imaging Reporting and Data System (PI-RADS) established minimum technical requirements and guidelines aimed to improve image quality and reduce variability [10]. To further address these obstacles, the Prostate Imaging Quality (PI-QUAL) scoring system was introduced in 2020 [11]. Since image quality is often “in the eye of the beholder,” the literature reflects mixed findings on how mpMRI quality impacts the accuracy of EPE prediction [12–15]. A more standardized method of assessing image quality might be useful in determining its importance in diagnosis.

Prostate MRI quality evaluations are typically conducted by radiologists using either a general assessment approach or specific criteria like PI-QUAL. However, these methods can be subject to variability due to their inherently qualitative nature, presenting a challenge in maintaining consistent standards [16, 17]. In this context, deep learning-based artificial intelligence (AI) emerges as a promising tool for the objective assessment of prostate MRI scans, potentially overcoming the variability of human evaluations. Recent studies have shown AI's capability in accurately assessing the quality of T2WI [18] and identifying the impact of AI-based quality evaluation on the performance of MRI targeted biopsies [19]. Despite these advancements, the clinical impact of prostate MRI quality, especially AI-based evaluations, remains largely underexplored. Therefore, this study aims to investigate the impact of T2WI quality on EPE detection using a deep learning-based AI algorithm.

## Materials and methods

### Patient population

**Fig. 1:**
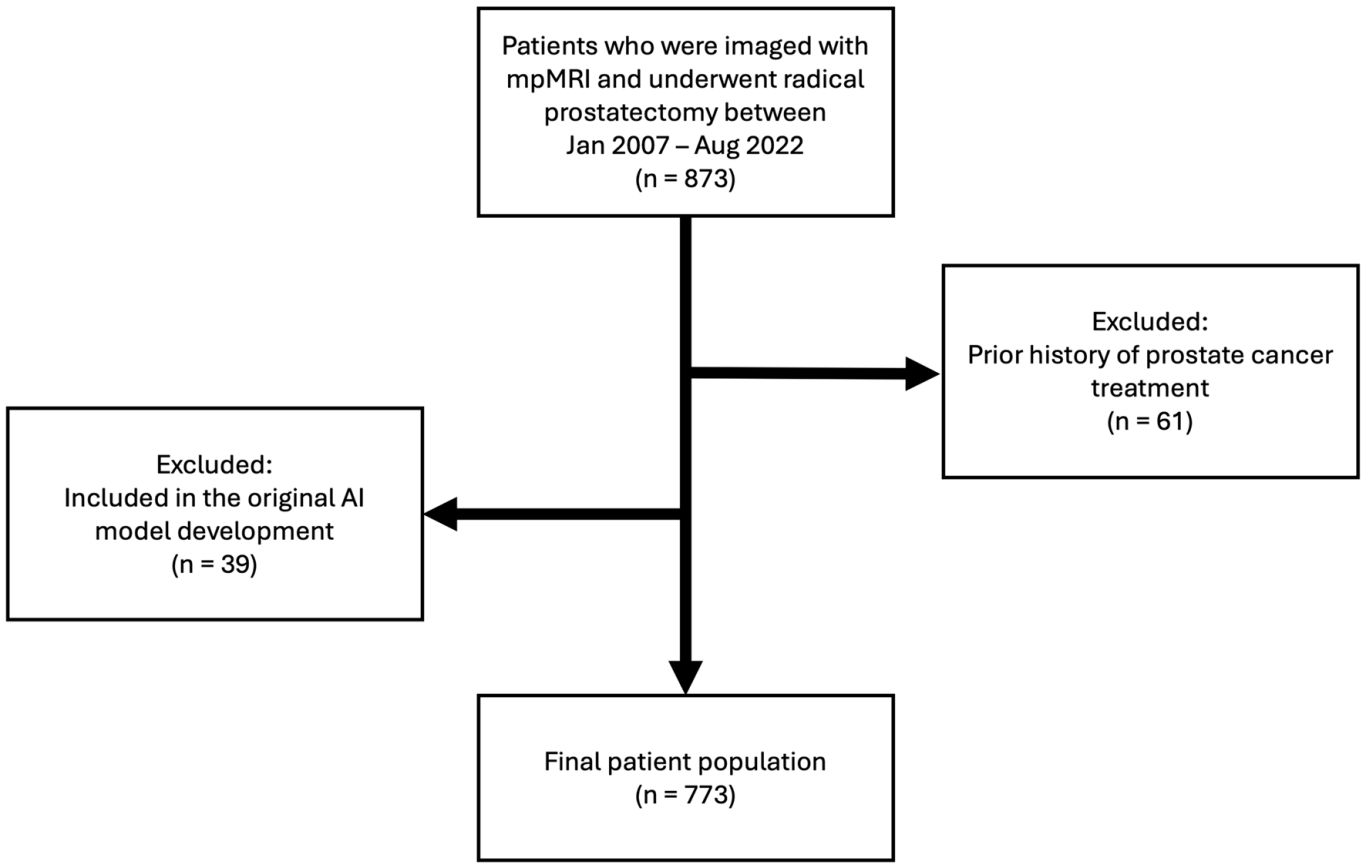
Patient flow diagram of the study. mpMRI multiparametric MRI, AI artificial intelligence

This HIPAA-compliant retrospective study was approved by the Institutional Review Board, and written informed consent was obtained from all patients (ClinicalTrials.gov identifier: NCT03354416, NCT00026884, and NCT02594202). A prospectively maintained institutional database was retrospectively queried for consecutive patients who were imaged with mpMRI and subsequently underwent RP at an academic center from June 2007 to August 2022 (Fig. 1). Patients were excluded from the study if they had received previous prostate cancer treatment (N = 61), or if they were part of the initial cohort used to train the AI algorithm (N = 39). The results from a subset of 604 patients were previously published in a study that evaluated MRI-based staging in predicting biochemical recurrence of prostate cancer after RP [20].

### Image acquisition and evaluation

MRI examinations were performed on two 3 T scanners (Achieva 3.0 T TX scanner or Ingenia Elition 3.0 T X, Philips Healthcare, Best, Netherlands), using a 16-channel surface coil (SENSE, Philips Healthcare, Best, Netherlands), with (*n* = 587) or without (*n* = 186) an endorectal coil (BPX-30, Medrad, PA, USA). Before imaging, each patient was instructed to undergo an enema to reduce rectal air. T2W turbo-spin-echo MRI, high b-value echo-planar diffusion-weighted imaging (DWI), and gradient recalled echo dynamic contrast-enhanced (DCE) sequences were obtained. Full image acquisition parameters are summarized in Supplemental Table 1.

From 2010 to 2022, scans were prospectively evaluated during clinical readouts by one genitourinary radiologist (B.T. with experience in prostate imaging since 2007). From 2007 to 2010, a different radiologist was responsible for interpreting the examinations and comprehensive cancer staging evaluations were not part of clinical workflow at the time. Therefore, for the current study, the aforementioned radiologist (B.T.) conducted retrospective interpretations of examinations during this period. EPE was assessed using the NCI EPE grading system [7]. The 3-point grading system was defined as follows: curvilinear contact length of 1.5 cm or capsular bulge and irregularity were grade 1, the presence of both features was grade 2, and frank capsular breach was grade 3. An EPE grading score ≥ 1 was considered as positive EPE call. Only the index lesions (i.e., those with highest PI-RADS category) per patient were considered for statistical analysis.

To assess inter-reader agreement of EPE, a subtest of MRI scans was evaluated by a second genitourinary radiologist (Y.M.L. with 10 years of experience in prostate cancer imaging) from a different institution. Eighty scans were assessed, consisting of 20 scans randomly selected for each of the four NCI EPE grades (grade 0, 1, 2, 3) based on the interpretations of the first reader. The second reader was blinded to clinical and pathological details, as well as to the first reader’s interpretations.

### Radical prostatectomy and histopathologic evaluation

Two urologists (P.A.P with 23 years of experience or S.G. with 5 years of experience) performed RP. Each surgical specimen was reviewed by one genitourinary pathologist (M.J.M. with 45 years of experience) during clinical workflow, blinded to the mpMRI results. Histopathologic evaluation was performed at RP according to the International Society of Urological Pathology (ISUP) consensus statement [21].

### T2W MR image quality assessment AI model

The previously published prostate image quality assessment AI model classified T2WI as high-quality (no quality distortions) versus low-quality (distortions present) [18]. A radiologist (B.T.) evaluated T2WI quality as high- or low-quality based on general distortions (e.g., motion, noise, aliasing) and perceptual distortions (e.g., obscured delineation of the prostatic capsule, prostatic zones, external urethral sphincter, excess rectal gas). This radiologist's assessment was used as the ground truth to train the AI algorithm. The AI model can be found and accessed via the GitHub repository at: https://github.com/NIH-MIP/Prostate-MRI_T2W_Quality.

### Statistical analysis

Pearson's chi-square [22] and nonparametric Wilcoxon–Mann Whitney tests [23] were conducted to examine the differences in the distribution of categorical and continuous variables, respectively. Fisher’s exact tests [24] were performed to compare EPE detection metrics (i.e., sensitivity, specificity, positive predictive value [PPV], or negative predictive value [NPV]) between high- and low-quality images groups. The 95% confidence intervals (CIs) of the diagnostic metrics were obtained from 2000 bootstrap samples by random sampling on the patient-level. Receiver operating characteristic (ROC) curves were created, and the area under the ROC curve (AUC) was calculated. AUC between high- and low-quality images groups were compared using the Delong test for correlated ROC curves [25]. Univariable and multivariable logistic regressions with backward variable selection based on the Akaike information criterion were applied to correlate with pathologic EPE [26]. The unweighted and quadratically weighted Cohen's kappa were used to evaluate agreement between the two readers [27]. Kappa values were categorized as slight (0–0.20), fair (0.21–0.40), moderate (0.41–0.60), substantial (0.61–0.80), and excellent (0.81–1). All tests were two-sided and a P value of < 0.05 was considered statistically significant. Statistical analyses were performed using R software (version 4.2.1; R Foundation for Statistical Computing).

## Results

### Patient characteristics

The final study population consisted of 773 patients, with a median age of 61 (IQR 56–67) years. The median serum prostate-specific antigen level was 6.5 (IQR 4.6–10.2) ng/mL, and the median prostate-specific antigen density was 0.16 (IQR 0.10–0.26) ng/mL^2^. The median time interval between MRI and RP was 95 (IQR 51–158) days. At RP, 23% (180/773) of patients had pathologic EPE. On mpMRI, 41% (131/318) of positive EPE calls were found to have EPE at pathology. The distribution of NCI EPE grades on imaging was as follows: grade 0 accounted for 59% (455/773), grade 1 for 12% (95/773), grade 2 for 18% (137/773), and grade 3 for 11% (86/773). Detailed demographic and clinical characteristics of the study population are summarized in Table 1.

**Table 1:** Patient demographics and characteristics

Variables	All patients (*n* = 773)	Low-quality T2WI (*n* = 280)	High-quality T2WI (*n* = 493)	*P* value
Age (y)*	61 (56–67)	60 (55–65)	62 (57–68)	0.17
PSA level (ng/mL)*	6.5 (4.6–10.2)	6.2 (4.4–8.8)	6.9 (4.8–10.7)	< 0.01
Prostate volume (cm^3^)*	42 (32–55)	38 (30–51)	43 (33–58)	< 0.01
PSA density (ng/mL^2^)*	0.16 (0.10–0.26)	0.15 (0.10–0.23)	0.16 (0.10–0.26)	0.33
Highest PI-RADS category^a^				0.95
1	9 (1)	3 (1)	6 (1)	
2	12 (1)	4 (2)	8 (2)	
3	77 (12)	25 (10)	52 (12)	
4	211 (32)	75 (31)	136 (32)	
5	358 (54)	133 (55)	225 (53)	
NCI EPE grade				0.15
0	455 (59)	152 (54)	303 (61)	
1	95 (12)	37 (13)	58 (12)	
2	137 (18)	60 (21)	77 (16)	
3	86 (11)	31 (11)	55 (11)	
ISUP grade group on RP				0.81
1	60 (8)	25 (9)	35 (7)	
2	403 (52)	145 (52)	258 (52)	
3	81 (10)	32 (11)	49 (10)	
4	153 (20)	53 (19)	100 (20)	
5	75 (10)	25 (9)	50 (10)	
Pathologic EPE				0.83
No	593 (77)	216 (77)	377 (76)	
Yes	180 (23)	64 (23)	116 (24)	

Unless otherwise specified, data are numbers of patients. Numbers in parentheses indicate the percentages

*ISUP* International Society of Urological Pathology, *mpMRI* multiparametric MRI, *PSA* prostate-specific antigen, *PI-RADS* Prostate Imaging Reporting and Data Systems, *NCI* National Cancer Institute, *RP* radical prostatectomy, *EPE* extraprostatic extension, *T2WI* T2-weighted imaging

*Data are median values, with IQRs in parentheses

^a^Data are missing for 40 low-quality T2WI scans and 66 high-quality T2WI scans

### Inter-reader agreement

The two readers had fair to substantial agreement for evaluation of NCI EPE grade (*n* = 80, unweighted κ = 0.30 [95% CI 0.16–0.44]; weighted κ = 0.63 [95% CI 0.49–0.78]) and moderate agreement for evaluation of frank EPE (NCI EPE grade = 3 versus grade 0–2) on MRI (unweighted κ = 0.41 [95% CI 0.17–0.64]).

### AI T2W image quality assessment

**Fig. 2:**
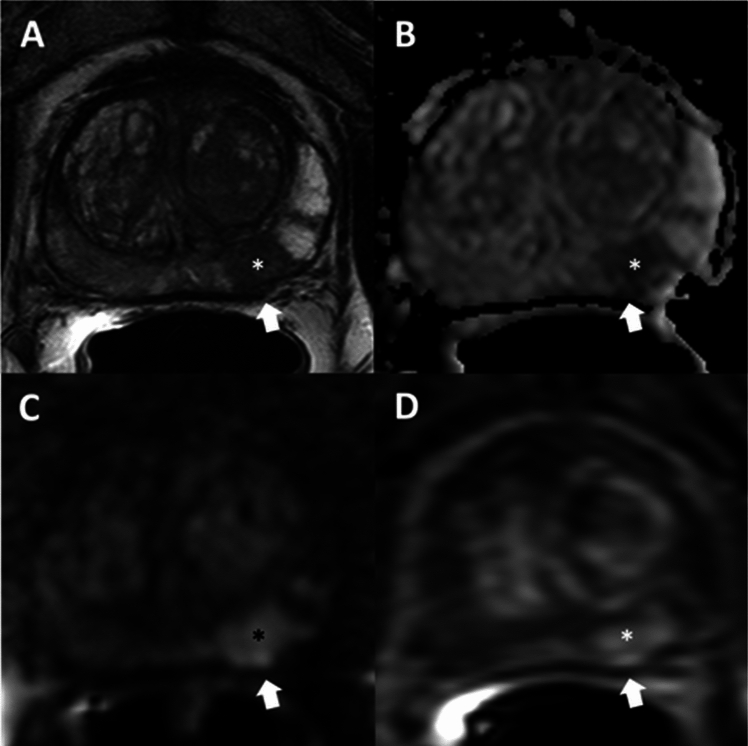
Multiparametric MRI of a 59-year-old patient with a serum prostate-specific antigen level of 6.1 ng/mL. AI classified T2WI as high-quality. The lesion (asterisk) was 1.5 cm in the left mid-base peripheral zone and assigned PI-RADS category 5. Slight capsular bulge was noted (NCI EPE grade = 1) (arrows). Radical prostatectomy showed Gleason score 9 (4 + 5) prostate adenocarcinoma with EPE, representing a true positive EPE call. Axial T2WI (**A**), apparent diffusion coefficient map (**B**), high b-value (b = 1500 s/mm^2^) diffusion weighted imaging (**C**), dynamic contrast enhanced imaging (**D**). AI artificial intelligence, T2WI T2-weighted imaging, PI-RADS Prostate Imaging Reporting and Data System, NCI National Cancer Institute, EPE extraprostatic extension

**Fig. 3:**
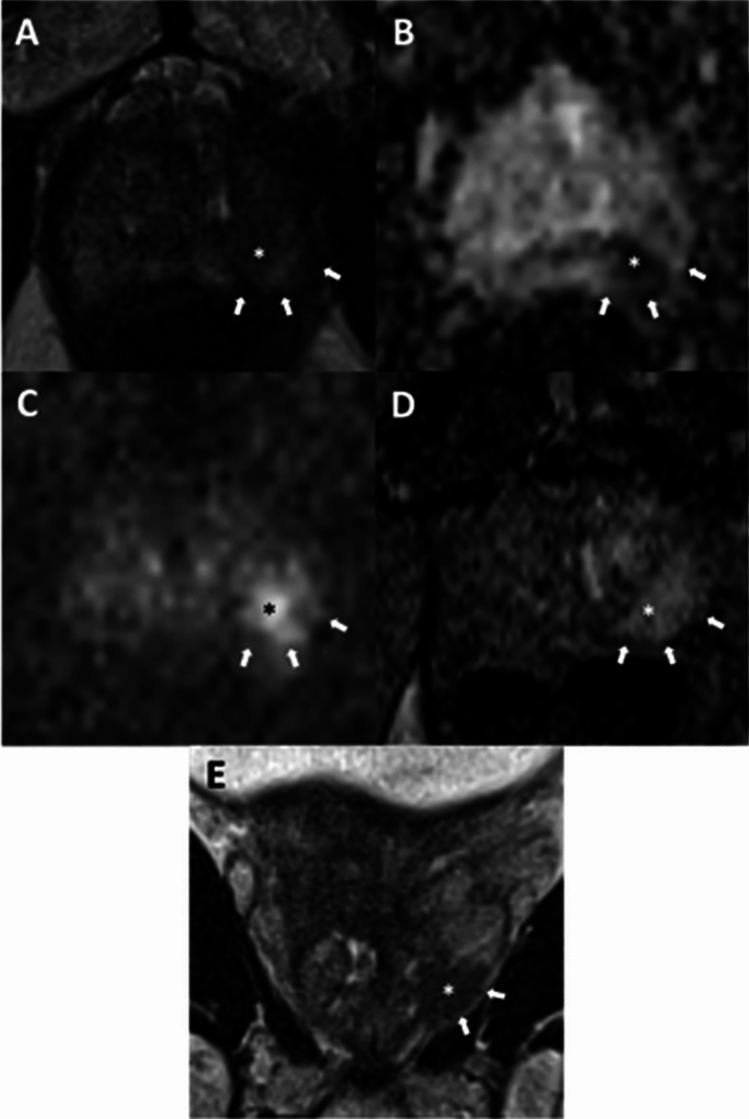
Multiparametric MRI of a 69-year-old patient with a serum prostate-specific antigen level of 8.2 ng/mL. T2WI had lower SNR and motion artifacts, whereas ADC map and high b-value DWI had lower SNR as well. AI classified T2WI as low-quality. The lesion (asterisk) was 1.7 cm in the left apical-base peripheral zone and assigned PI-RADS category 5. Long curvilinear contact length and capsular irregularity/bulge were noted (NCI EPE grade = 2) (arrows). Radical prostatectomy showed Gleason score 8 (4 + 4) prostate adenocarcinoma with no EPE, representing a false positive EPE call. Axial T2WI (**A**), ADC map (**B**), high b-value (b = 1500 s/mm^2^) DWI (**C**), dynamic contrast enhanced imaging (**D**), coronal T2WI (**E**). AI artificial intelligence, T2WI T2-weighted imaging, ADC apparent diffusion coefficient, DWI diffusion weighted imaging, PI-RADS Prostate Imaging Reporting and Data System, NCI National Cancer Institute, EPE extraprostatic extension, SNR = signal-to-noise ratio

The AI algorithm classified 493 of 773 (64%) T2WI as high-quality and 280 of 773 (36%) T2WI as low-quality. Examples of high- and low-quality scans are shown in Figs. 2 and 3, respectively.

### Diagnostic accuracy by image quality

The detection rate of EPE for NCI EPE grade 1 was 24% (23/95), grade 2 was 40% (55/137), and grade 3 was 62% (53/86). For NCI EPE grade ≥ 1, the sensitivity for predicting EPE across all scans was 73% (95% CI 66–79%), while specificity was 68% (95% CI 65–72%). The PPV and NPV were 41% (95% CI 38–45%) and 89% (95% CI 87–91%), respectively. For NCI EPE grade ≥ 2, the sensitivity was 60% (95% CI 52–67%), with a specificity of 81% (95% CI 77–84%), PPV of 48% (95% CI 43–54%), and NPV of 87% (95% CI 85–89%). For NCI EPE grade ≥ 3, overall sensitivity was 29% (95% CI 23–37%), while specificity was 94% (95% CI 92–96%). The PPV and NPV were 62% (95% CI 52–71%) and 82% (95% CI 80–83%), respectively. The detailed diagnostic measures for detecting EPE across NCI EPE grades are summarized in Table 2*.*

**Table 2:** Diagnostic measures for detecting EPE

Variables	All patients (*n* = 773)	Low-quality T2WI (*n* = 280)	High-quality T2WI (*n* = 493)	*P* value
NCI EPE grade ≥ 1				
Sensitivity	73 (66, 79) [131/180]	75 (63, 85) [48/64]	72 (62, 80) [83/116]	1
Specificity	68 (65, 72) [406/593]	63 (56, 69) [136/216]	72 (67, 76) [270/377]	0.03
PPV	41 (38, 45) [131/318]	38 (32, 43) [48/128]	44 (39, 49) [83/190]	0.30
NPV	89 (87, 91) [406/455]	89 (85, 93) [136/152]	89 (86, 92) [270/303]	1
NCI EPE grade ≥ 2				
Sensitivity	60 (52, 67) [108/180]	66 (53, 77) [42/64]	57 (47, 66) [66/116]	0.43
Specificity	81 (77, 84) [478/593]	77 (71, 83) [167/216]	52 (78, 86) [311/377]	0.13
PPV	48 (43, 54) [108/223]	46 (39, 54) [42/91]	50 (43, 57) [66/132]	0.59
NPV	87 (85, 89) [478/550]	88 (84, 91) [167/189]	86 (83, 89) [311/361]	0.51
NCI EPE grade ≥ 3				
Sensitivity	29 (23, 37) [53/180]	28 (17, 41) [18/64]	30 (22, 39) [35/116]	0.86
Specificity	94 (92, 96) [560/593]	94 (90, 97) [203/216]	95 (92, 97) [357/377]	0.71
PPV	62 (52, 71) [53/86]	58 (42, 73) [18/31]	63 (51, 74) [35/55]	0.65
NPV	82 (80, 83) [560/687]	82 (79, 84) [203/249]	81 (80, 83) [357/438]	1
AUC	0.74 (0.70, 0.78)	0.74 (0.67, 0.81)	0.75 (0.70, 0.80)	0.88

Data in parentheses are 95% confidence intervals and data in brackets are numerator/denominator

*NCI* National Cancer Institute, *EPE* extraprostatic extension, *T2WI* T2-weighted imaging, *PPV* positive predictive value, *NPV* negative predictive value, *AUC* area under the receiver operating characteristic curve

AUC, sensitivity, PPV, and NPV across different EPE grades did not demonstrate significant difference attributable to imaging quality. For NCI EPE grade ≥ 1, the specificity was significantly higher in the high-quality T2WI group compared to the low-quality group (72% [95% CI 67–76%] vs 63% [95% CI 56–69%], *P* = 0.03). Additionally, MRI and pathology mismatches (i.e., positive MRI call [EPE grade ≥ 1] but negative pathology) were observed in 31% of total cases. The mismatch was slightly more prevalent in the low-quality T2WI subgroup at 34% compared to 28% in the high-quality subgroup, approaching statistical significance (*P* = 0.09).

An additional analysis done to test the impact of pre-MRI biopsy status on EPE prediction revealed no difference for MRI’s prediction performance between patients who had MRI before and after prostate biopsy (Supplemental table 2). Finally, we evaluated the impact of use of ERC on quality assessments and EPE predictions. We observed no impact of use of ERC on image quality and EPE predictions (Supplemental Table 3).

### Univariable and multivariable logistic regression analysis for EPE prediction

Clinical and pathologic variables, imaging features, and image quality were assessed in a univariable logistic regression model for predicting pathologic EPE. Prostate-specific antigen (odds ratio [OR] 1.07, 95% CI 1.05–1.09; *P* < 0.001), prostate-specific antigen density (OR 20.33, 95% CI 8.08–54.29; *P* < 0.001), ISUP grade 4 (OR 3.70, 95% CI 1.95–7.40; *P* < 0.001) and 5 (OR 9.75, 95% CI 4.72–21.17; *P* < 0.001), and peripheral zone index lesion (OR 1.52, 95% CI 1.02–2.29; *P* = 0.04) were significant predictors of EPE on histopathology. Increasing NCI EPE grades were associated with a stepwise increase in the OR of predicting pathologic EPE (Table 3). Compared to patients without evidence of EPE on MRI, those with NCI EPE grade 1 on high-quality images showed a significant association with pathologic EPE (OR 3.44, 95% CI 1.78–6.43; *P* < 0.001). Conversely, patients with NCI EPE grade 1 on low-quality images did not demonstrate a significant association with pathologic EPE (OR 1.60, 95% CI 0.58–3.79; *P* = 0.32).

**Table 3:** Univariable and multivariable logistic regression model for pathologic EPE risk prediction

	Univariable logistic regression model			Multivariable logistic regression model		
Variables	Odds ratio	95% CI	*P* value	Odds ratio	95% CI	*P* value
Clinical information						
Age	1.02	1.00–1.05	0.051			
PSA	1.07	1.05–1.09	< 0.001			
PSAD	20.33	8.08–54.29	< 0.001	3.61	1.39–10.16	0.01
PI-RADS category						
PI-RADS ≤ 2	Ref	–	–			
PI-RADS 3	0.50	0.06–10.53	0.56			
PI-RADS 4	1.64	0.31–30.44	0.64			
PI-RADS 5	6.64	1.30–121.2	0.07			
Biopsy ISUP categories						
ISUP grade 1	Ref	–	–			
ISUP grade 2	1.27	0.69–2.50	0.46			
ISUP grade 3	1.26	0.60–2.71	0.55			
ISUP grade 4	3.70	1.95–7.40	< 0.001	2.09	1.30–3.35	0.002
ISUP grade 5	9.75	4.72–21.17	< 0.001	5.13	2.77–9.58	< 0.001
Index lesion location						
Posterior location	1.50	0.99–2.31	0.06			
Peripheral zone	1.52	1.02–2.29	0.04			
NCI EPE grade with image quality						
Grade 0 (low- and high-quality)	Ref	–	–			
Grade 1 low-quality	1.60	0.58–3.79	0.32	1.76	0.63–4.24	0.24
Grade 1 high-quality	3.44	1.78–6.43	< 0.001	3.05	1.54–5.86	< 0.001
Grade 2 low-quality	5.52	3.03–10.00	< 0.001	4.25	2.21–8.05	< 0.001
Grade 2 high-quality	5.58	3.23–9.62	< 0.001	4.99	2.80–8.86	< 0.001
Grade 3 low-quality	11.77	5.34–25.33	< 0.001	6.63	2.89–15.44	< 0.001
Grade 3 high-quality	14.50	7.85–27.50	< 0.001	10.63	5.55–20.82	< 0.001

*CI* confidence interval, *PSA* prostate-specific antigen, *PSAD* prostate-specific antigen density, *PI-RADS* Prostate Imaging Reporting and Data System, *ISUP* International Society of Urological Pathology, *NCI* National Cancer Institute, *EPE* extraprostatic extension

In the multivariable logistic regression model, prostate-specific antigen density (OR 3.61, 95% CI 1.39–10.16; *P* = 0.01) and ISUP grade 4 (OR 2.09, 95% CI 1.30–3.35; *P* = 0.002) and grade 5 (OR 5.13, 95% CI 2.77–9.58; *P* < 0.001) remained significant predictors. NCI EPE grade 1 with high-quality images was significantly associated with pathologic EPE (OR 3.05, 95% CI 1.54–5.86; *P* < 0.001), whereas NCI EPE grade 1 with low-quality images was not associated with pathologic EPE (OR 1.76, 95% CI 0.63–4.24; *P* = 0.24). For NCI EPE grade 2, both low-quality images (OR 4.25, 95% CI 2.21–8.05; *P* < 0.001) and high-quality images (OR 4.99, 95% CI 2.80–8.86; *P* < 0.001) showed significant association with pathologic EPE. Similarly, for NCI EPE grade 3, both low-quality images (OR 6.63, 95% CI 2.89–15.44; *P* < 0.001) and high-quality images (OR 10.63, 95% CI 5.55–20.82; *P* < 0.001) were significantly associated with pathologic EPE.

## Discussion

The detection of EPE, a critical indicator of prostate cancer aggressiveness, is crucial for guiding treatment decisions and surgical strategies. However, the interpretation of mpMRI and detection of EPE are not without challenges, partly due to the variability in image quality and the subjective nature of radiological assessments. AI holds the potential to support physicians in objectively and swiftly evaluating the quality of MRI scans. Thus, this study investigated the impact of T2W image quality, assessed with a previously developed AI algorithm, on the detection of EPE in patients undergoing RP. While image quality did not significantly affect sensitivity, PPV, or NPV, a notable improvement in specificity for EPE detection was observed for high-quality T2WI in NCI EPE grade ≥ 1 (72% vs. 63%, *P* = 0.03). Additionally, both univariable and multivariable analyses showed that NCI EPE grade 1 high-quality images demonstrated a stronger association with pathologic EPE than low-quality images.

The current literature reports a wide range of accuracy in predicting pathologic EPE, which may stem from variations in measurement metrics and modality of assessment of radiologic EPE [28–30]. In our study, we evaluated the presence of radiologic EPE via the NCI EPE grading system, which has the benefits of simplicity and standardization. Our results on the prediction of EPE using mpMRI underscored its high specificity yet modest sensitivity. This aligns with findings from a meta-analysis which evaluated the diagnostic performance of mpMRI for identifying EPE, with a pooled sensitivity and specificity of 0.57 and 0.91, respectively [31]. Another meta-analysis investigating the NCI EPE grading system, reported a hierarchical summary AUC of 0.82 for EPE prediction [32], which is consistent with the AUC of 0.74 in our study.

As prostate MR image quality is a fairly new research area, most studies have focused on the impact of PI-QUAL score on EPE prediction [12–15]. In a retrospective study with 146 patients, Coelho et al. [14] found that PI-QUAL score does not affect the overall accuracy of EPE prediction. Specifically, the AUC was 0.75 for images with a PI-QUAL score of 3 or less, and 0.705 for images with a PI-QUAL score of 4 or higher. PI-QUAL score did not show correlation with EPE prediction in both univariable and multivariable analyses. Due to the limited sample size (*n* = 146), statistical significance for certain diagnostic measures, such as specificity, was not evaluated in their study. With a much larger study population, the current study demonstrated a statistically significant difference in specificity for NCI EPE grade ≥ 1. We also found that high-quality images were associated with higher ORs for predicting EPE across all grades. Notably, NCI EPE grade 1 with low-quality images was not a significant predictor for pathologic EPE on multivariable analysis. These findings suggest that AI-based imaging quality assessments could significantly influence patient risk stratification based on T2WI quality, thus enabling more personalized therapeutic strategies. In another retrospective study with 105 patients, Ponsiglione et al. found that specifically for EPE grade 3, accuracy was higher in studies with PI-QUAL ≥ 4 compared to with PI-QUAL < 4 (0.849 vs. 0.564, *P* = 0.001) [13]. This contrasts with our results. However, it’s important to note that PI-QUAL scoring system utilizes all mpMRI sequences, whereas the AI model we used in our study focused only on T2WI, which is recognized as the most critical anatomic pulse sequence for detecting EPE. This distinction highlights the importance of pulse sequence-specific analysis in enhancing the precision of EPE prediction.

Research regarding automated AI for evaluating the quality of prostate MR images is still emerging, with few studies available thus far [18, 33, 34]. Nonetheless, the findings to date are encouraging. One AI model demonstrated near-perfect accuracy in its testing phase [33]. The AI model used in our study achieved an accuracy of 84.7% in 1046 scans during its development phase [18]. This level of accuracy establishes the potential of AI to significantly enhance the assessment of image quality, setting a solid foundation for further clinical applications. For example, a study using this AI algorithm to evaluate the impact of T2W image quality on prostate cancer detection rates found that higher quality T2WIs were associated with higher rates of clinically significant cancer detection for PI-RADS 4 lesions [19]. Looking ahead, it's plausible that AI-driven image quality assessment will be seamlessly integrated into clinical and research workflows, ensuring uniform image quality. Furthermore, this AI model has the potential for real-time application during scans, offering prompt assistance to technologists in making informed decisions about the necessity of rescans [16].

Our study has some limitations. Its retrospective nature and reliance on a single institution's dataset may introduce selection bias. The interpretation of MRI scans was conducted by one radiologist, and RP and pathology assessments were performed by specialists in their respective areas. However, all of these were done as part of a clinical routine practice and not in a research manner, which mirrors the real life scenario in academic clinical practice setting. The study population consisted of patients undergoing RP, which might include different clinical or imaging characteristics from non-surgical populations. The results of the multireader analysis suggested some interobserver variability. These factors might limit the generalizability of the findings. Future research should aim to validate these results in a multicenter study, incorporating a larger and more diverse patient cohort. Of note, the high-quality T2WI group had significantly higher prostate specific antigen levels and prostate volumes compared to the low-quality group. These clinical variables could potentially influence the assessment of image quality and the evaluation of EPE. Additionally, the univariable and multivariable analyses suggested a trend of increasing ORs for predicting pathologic EPE with higher image quality. However, the wide overlap of CIs, particularly for EPE grades 2 and 3, indicates that while the ORs are higher for high-quality images, the clinical significance may require careful interpretation. Moreover, the AI model evaluated in this study is limited to assessing the quality of T2WI. In practice, radiologists may utilize additional sequences, including DWI and DCE MRI, for evaluation of EPE. Our group is actively developing AI models to evaluate the quality of these functional MRI pulse sequences.

In conclusion, this study demonstrated the significant impact of T2W image quality, assessed by an AI algorithm, on the detection of EPE in patients undergoing RP. The findings revealed that high-quality T2WI significantly improved the specificity for NCI EPE grade ≥ 1, and that NCI EPE grade 1 was associated with pathologic EPE only when high-quality images were utilized. Given the challenges in EPE detection and the variability in MRI quality, integrating AI-based image quality assessments could provide a promising solution for more tailored and standardized prostate cancer evaluations.

### Supplementary Information

Below is the link to the electronic supplementary material.

Supplementary file1 (DOCX 17 kb)

## Data Availability

Not applicable.

## Code availability

https://github.com/NIH-MIP/Prostate-MRI_T2W_Quality.
