# Supplementary material for: Deep learning-based image quality assessment: impact on detection accuracy of prostate cancer extraprostatic extension on MRI
Source: Abdom Radiol (NY). 2024 Jul 3;49(8):2891–901. doi: 10.1007/s00261-024-04468-5 (PMC11300622; doi:10.1007/s00261-024-04468-5)
Supplement: Supplementary file 1 — Supplementary file1 (DOCX 17 kb) [file 261_2024_4468_MOESM1_ESM.docx]

**SUPPLEMENTAL MATERIALS**

***Endorectal Coil Analysis***

A total of 773 examinations were conducted, with 587 using an ERC and 186 without. The AI algorithm classified 364 scans (62.2%) in the ERC group as high-quality and 221 scans (37.8%) as low-quality. In the non-ERC group, 129 scans (68.7%) were classified as high-quality and 59 scans (31.4%) as low-quality. No statistically significant difference was observed between the quality distributions of these two groups (*P* = 0.11). Furthermore, no significant difference was found in the diagnostic metrics for EPE between the two groups. Detailed diagnostic measures for detecting EPE across NCI EPE grades between the ERC and non-ERC groups are summarized in Supplemental Table 3.

**Supplemental Table 1:** Image acquisition parameters

| **Parameters** | **With ERC (***n* = 587**)** | | | **Without ERC (***n* =186**)** | | |
| --- | --- | --- | --- | --- | --- | --- |
|  | **T2WI** | **DWI**  **(b = 2000 s/mm^2^)** | **DCE*** | **T2WI** | **DWI (b=1500 s/mm^2^)** | **DCE*** |
| Field of view (mm) | 140 × 140 | 140 × 140 | 262 × 262 | 180 × 180 | 140 × 140 | 262 × 262 |
| Acquisition matrix | 304 × 234 | 76 × 78 | 188 × 96 | 320 × 216 | 64 × 62 | 176 × 66 |
| Repetition time (msec) | 4434 | 6987 | 3.7 | 3686 | 7218 | 3.7 |
| Echo time (msec) | 120 | 52 | 2.3 | 120 | 47 | 2.3 |
| Flip angle (degrees) | 90 | 90 | 8.5 | 90 | 90 | 8.5 |
| Section thickness (mm) | 3 | 3 | 3 | 3 | 3 | 3 |
| Image reconstruction matrix (pixels) | 512 × 512 | 256 × 256 | 256 × 256 | 512 × 512 | 256 × 256 | 256 × 256 |
| Reconstruction voxel imaging resolution (mm/pixel) | 0.27 × 0.27 × 3 | 0.55 × 0.55 × 2.73 | 1.02 × 1.02 × 3 | 0.35 × 0.35 × 3 | 1.09 × 1.09 × 3 | 1.02 × 1.02 × 3 |
| Time for acquisition (min:sec) | 2:48 | 3:50 | 5:16 | 4:48 | 6:08 | 5:16 |
| Echo train length | 20 | 20 | 20 | 20 | 20 | 20 |
| Number of Averages | 1 | 10 |  | 3 | 2 |  |

Note. — Apparent diffusion coefficient calculation was based on three evenly spaced acquired b-values (0-600 s/mm^2^). ERC = Endorectal coil, T2WI = T2-weighted imaging, DWI = Diffusion-weighted imaging, DCE = Dynamic contrast enhanced imaging.

* DCE MRI temporal resolution is 5.6 seconds.

**Supplemental Table 2:** Diagnostic Measures for Detecting EPE using Pre- or Post-biopsy MRI

| Variables* | Pre-biopsy  (*n* = 591) | Post-biopsy  (*n* = 122) | *P* Value |
| --- | --- | --- | --- |
| NCI EPE grade ≥1 |  |  |  |
| Sensitivity | 100/136 (74) | 22/28 (79) | 0.64 |
| Specificity  PPV  NPV | 319/456 (70)  100/237 (42)  319/355 (90) | 60/94 (64)  22/56 (39)  60/66 (91) | 0.27  0.76  1 |
| NCI EPE grade ≥2 |  |  |  |
| Sensitivity  Specificity  PPV | 83/136 (61)  369/456 (81)  83/170 (49) | 19/28 (68)  74/94 (79)  19/39 (49) | 0.53  0.67  1 |
| NPV | 369/422 (87) | 74/83 (89) | 0.85 |
| NCI EPE grade ≥3 |  |  |  |
| Sensitivity  Specificity  PPV | 39/136 (39)  433/456 (95)  39/62 (63) | 12/28 (43)  86/94 (91)  12/20 (60) | 0.18  0.22  1 |
| NPV | 433/530 (82) | 86/102 (84) | 0.58 |

Note. — Unless otherwise specified, data are numbers of patients. Numbers in parentheses indicate the percentages. NCI = National Cancer Institute, EPE = extraprostatic extension, PPV = positive predictive value, NPV = negative predictive value.

*Biopsy date data point was missing in 60 patients.

**Supplemental Table 3:** Diagnostic Measures for Detecting EPE with or without ERC

| Variables | With ERC  (*n* = 587) | Without ERC  (*n* = 186) | *P* Value |
| --- | --- | --- | --- |
| NCI EPE grade ≥1 |  |  |  |
| Sensitivity | 103/136 (76) | 28/44 (64) | 0.12 |
| Specificity  PPV  NPV | 300/451(67)  103/254 (41)  300/333 (90) | 106/142 (75)  28/64 (44)  106/122 (87) | 0.08  0.67  0.39 |
| NCI EPE grade ≥2 |  |  |  |
| Sensitivity  Specificity  PPV | 83/136 (61)  359/451 (80)  83/175 (47) | 25/44 (57)  119/142 (84)  25/48 (52) | 0.72  0.33  0.63 |
| NPV | 359/412 (87) | 119/138 (86) | 0.77 |
| NCI EPE grade ≥3 |  |  |  |
| Sensitivity  Specificity  PPV | 41/136 (30)  428/451 (95)  41/64 (64) | 12/44 (27)  132/142 (93)  12/22 (55) | 0.85  0.40  0.46 |
| NPV | 428/523 (82) | 132/164 (80) | 0.73 |

Note. — Unless otherwise specified, data are numbers of patients. Numbers in parentheses indicate the percentages. NCI = National Cancer Institute, EPE = extraprostatic extension, ERC = endorectal coil, PPV = positive predictive value, NPV = negative predictive value.
